# Longitudinal atrophy in prodromal dementia with Lewy bodies points to cholinergic degeneration

**DOI:** 10.1093/braincomms/fcac013

**Published:** 2022-02-07

**Authors:** Kejal Kantarci, Zuzana Nedelska, Qin Chen, Matthew L. Senjem, Christopher G. Schwarz, Jeffrey L. Gunter, Scott A. Przybelski, Timothy G. Lesnick, Walter K. Kremers, Julie A. Fields, Jonathan Graff-Radford, Rodolfo Savica, David Jones, Hugo Botha, David S. Knopman, Val Lowe, Neill R. Graff-Radford, Melissa M. Murray, Dennis W. Dickson, R. Ross Reichard, Clifford R. Jack, Ronald C. Petersen, Tanis J. Ferman, Bradley F. Boeve

**Affiliations:** 1 Department of Radiology, Mayo Clinic, Rochester, MN, USA; 2 Department of Neurology, Charles University, Prague, Czech Republic; 3 Department of Neurology, West China Hospital of Sichuan University, Chengdu, Sichuan, China; 4 Department of Health Sciences Research, Mayo Clinic, Rochester, MN, USA; 5 Department of Psychology and Psychiatry, Mayo Clinic, Rochester, MN, USA; 6 Department of Neurology, Mayo Clinic, Rochester, MN, USA; 7 Department of Neurology, Mayo Clinic, Jacksonville, FL, USA; 8 Department of Laboratory Medicine and Pathology, Mayo Clinic, Rochester, MN, USA; 9 Department of Laboratory Medicine and Pathology, Mayo Clinic, Jacksonville, FL, USA; 10 Department of Psychology and Psychiatry, Mayo Clinic, Jacksonville, FL, USA

**Keywords:** mild cognitive impairment, dementia with Lewy bodies, MRI, atrophy, prodromal DLB

## Abstract

Mild cognitive impairment with the core clinical features of dementia with Lewy bodies is recognized as a prodromal stage of dementia with Lewy bodies. Although grey matter atrophy has been demonstrated in prodromal dementia with Lewy bodies, longitudinal rates of atrophy during progression to probable dementia with Lewy bodies are unknown. We investigated the regional patterns of cross-sectional and longitudinal rates of grey matter atrophy in prodromal dementia with Lewy bodies, including those who progressed to probable dementia with Lewy bodies. Patients with mild cognitive impairment with at least one core clinical feature of dementia with Lewy bodies (mean age = 70.5; 95% male), who were enrolled in the Mayo Clinic Alzheimer’s Disease Research Center and followed for at least two clinical evaluations and MRI examinations, were included (*n* = 56). A cognitively unimpaired control group (*n* = 112) was matched 2:1 to the patients with mild cognitive impairment by age and sex. Patients either remained stable (*n* = 28) or progressed to probable dementia with Lewy bodies (*n* = 28) during a similar follow-up period and pathologic confirmation was available in a subset of cases (*n* = 18). Cross-sectional and longitudinal rates of grey matter atrophy were assessed using voxel-based and atlas-based region of interest analyses. At baseline, prodromal dementia with Lewy bodies was characterized by atrophy in the nucleus basalis of Meynert both in those who remained stable and those who progressed to probable dementia with Lewy bodies (*P* < 0.05 false discovery rate corrected). Increase in longitudinal grey matter atrophy rates were widespread, with greatest rates of atrophy observed in the enthorhinal and parahippocampal cortices, temporoparietal association cortices, thalamus and the basal ganglia, in mild cognitive impairment patients who progressed to probable dementia with Lewy bodies at follow-up (*P* < 0.05 false discovery rate corrected). Rates of inferior temporal atrophy were associated with greater rates of worsening on the clinical dementia rating–sum of boxes. Seventeen of the 18 (94%) autopsied cases had Lewy body disease. Results show that atrophy in the nucleus basalis of Meynert is a feature of prodromal dementia with Lewy bodies regardless of proximity to progression to probable dementia with Lewy bodies. Longitudinally, grey matter atrophy progresses in regions with significant cholinergic innervation, in alignment with clinical disease progression, with widespread and accelerated rates of atrophy in patients who progress to probable dementia with Lewy bodies. Given the prominent neurodegeneration in the cholinergic system, patients with prodromal dementia with Lewy bodies may be candidates for cholinesterase inhibitor treatment.

## Teaser

This study demonstrated that prodromal dementia with Lewy bodies is characterized by atrophy in the nucleus basalis of Meynert. Longitudinally, grey matter atrophy progressed in regions with significant cholinergic innervation, in alignment with clinical disease progression, with accelerated rates of atrophy in patients who progressed to dementia with Lewy bodies.

## Graphical Abstract

**Graphical abstract:**
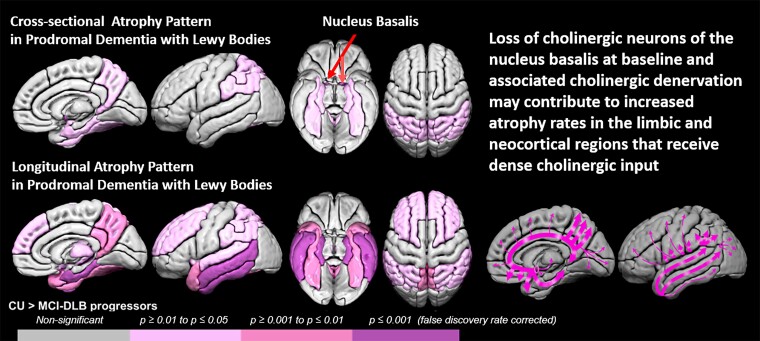

## Introduction

Based on the evidence that was available in 2020, the prodromal dementia with Lewy bodies (DLB) Diagnostic Study Group published the research criteria for the diagnosis of prodromal DLB.^1^ The prodromal phase of DLB includes three categories of presentations: (i) mild cognitive impairment (MCI)-onset, (ii) delirium-onset and (iii) psychiatric-onset, whereas evidence from the MCI-onset category of prodromal DLB was sufficient to propose formal criteria for prodromal DLB, the evidence from the delirium-onset and psychiatric-onset presentations were found to be insufficient.^1^ Thus, MCI with the core clinical features of DLB is now recognized as a prodromal stage of DLB (MCI-LB).

MCI-LB may be present years before probable DLB becomes evident. Non-amnestic MCI along with hippocampal preservation on MRI has been shown to be a strong predictor of progression from MCI-LB to probable DLB versus Alzheimer’s disease dementia.^2,3^ Cross-sectional structural MRI studies have demonstrated atrophy in insula, temporal and cingulate cortices, and the nucleus basalis of Meynert in MCI-LB; ^4–8^ however, clinical progression of MCI patients to probable DLB, as well as rates of longitudinal of atrophy during progression to probable DLB are unknown.

Patients with probable DLB are characterized by increased rates of global brain atrophy and cortical atrophy on longitudinal structural MRI compared to their cognitively unimpaired (CU) peers.^9–12^ Furthermore, regional grey matter atrophy rates are associated with clinical disease progression in both patients with clinically diagnosed probable DLB^13^ and autopsy-confirmed Lewy body disease,^12^ indicating that neurodegeneration is an important component of DLB pathophysiology. Determining the cross-sectional and longitudinal patterns of atrophy in MCI-LB and particularly in MCI-LB who progressed to probable DLB would provide an opportunity for detection of early neurodegeneration in patients with DLB at the prodromal stage. In addition, determining the topographic pattern and time course of structural MRI changes in MCI-LB may provide insights into the clinical evolution of DLB starting from the earliest stages.

Objectives of the present study were 2-fold: (i) to assess the pattern of cross-sectional and longitudinal rates of grey matter atrophy in MCI patients with at least one clinical core feature of DLB, (i.e. MCI-LB) compared with CU controls; (ii) to determine whether the rates of grey matter atrophy are associated with clinical disease progression in MCI-LB.

## Materials and methods

### Participants

Patients included those with MCI and at least one core clinical feature of DLB (i.e. parkinsonism, fluctuations, visual hallucinations, RBD), enrolled in the Mayo Clinic Alzheimer’s Disease Research Center (ADRC) between October 2005 and December 2017 and followed with at least two clinical evaluations and MRI exams (*n* = 56). Although the recently published research criteria for prodromal DLB^1^ was not used for inclusion at the time of enrolment to this prospective study, in retrospect 47 (84%) of the patients with MCI met the research criteria for probable prodromal DLB and the remaining 9 (16%) met the research criteria for possible prodromal DLB.

Mayo Clinic ADRC participants are followed prospectively with approximately annual clinical evaluations and MRI. To include all MCI-DLB participants and to keep the follow-up interval consistent between the MCI-DLB patients who remained stable (MCI-LB stable) and MCI-DLB patients who progressed to probable DLB (MCI-LB progressor), we included the last two evaluations prior to and at the time of progression to DLB in MCI-LB progressors (*n* = 28) and last two evaluations in MCI-LB stables (*n* = 28). The CU adults included as controls (CU; *n* = 112) were participants of the Mayo Clinic Study of Aging (MCSA), a prospective population-based study of aging.^14^ CU had at least two clinical evaluations and MRI examinations and were matched to MCI-LB 2:1 on age at baseline and sex.

### Clinical evaluation

Diagnosis of MCI was made according to the criteria by Petersen *et al*.,^15,16^ and diagnosis of probable DLB was made according to the DLB Consortium Criteria.^17,18^ Clinical Dementia Rating–Sum of Boxes (CDR–SOB) was used to determine clinical disease severity. Assessments for the clinical features of DLB were detailed in previous reports from the ADRC cohorts.^14,19^ Briefly, the 4-item Mayo Fluctuations Scale scores of 3 or 4^20^ were used to identify the presence of fluctuations. Visual hallucinations were characterized by being fully formed and not restricted to a single episode or related to another medical issue, or treatment. A history of probable REM sleep behaviour disorder (pRBD) was based on the International Classification of Sleep Disorders-II diagnostic criteria.^21^ Presence of parkinsonism was based on two of the four cardinal features (bradykinesia, rigidity, tremor and postural instability). The Unified Parkinson’s Disease Rating Scale-III (UPDRS-III) score^22^ was used to quantify the severity of motor impairment.

Informed consent was obtained from all participants and/or their proxies for participation in this study. All procedures were approved by the Mayo Clinic Institutional Review Board.

### MRI acquisition and analysis

All MRIs were performed at 3T with an 8-channel phased array head coil (GE Healthcare, Milwaukee, WI, USA). A 3D high-resolution T_1_-weighted magnetization-prepared rapid gradient echo acquisition was performed with repetition time/echo time/inversion time = 2300/3/900 ms, flip angle 8°, voxel resolution 1.2×1×1 mm.

T_1_-weighted MRI were tissue-class segmented and corrected for B0 inhomogeneities using Unified Segmentation^23^ in SPM12 (www.fil.ion.ucl.ac.uk/spm) with population-optimized templates and settings from the Mayo Clinic Adult Lifespan Template (MCALT; https://www.nitrc.org/projects/mcalt/). Tissue volumes were calculated by summing voxel-wise probabilities within each of 28 cortical and subcortical region of interest (ROI) labels from the an in-house atlas of the substantia innominata, propagated using ANTS Symmetric Normalization,^24^ with right and left hemispheric values averaged as previously described.^25^ We then estimated cortical thickness from these segmentations using ANTS DiReCT, also as previously described.^26^

We used a fully automated in-house developed image processing pipeline, named tensor-based morphometry with symmetric normalization (TBM-SyN), to compute the changes in cortical volume over time in each participant.^27,28^ Briefly, the steps in the TBM-SyN pipeline were as follows. First, for each participant, both of their longitudinal T_1_-weighted structural MRI scans were iteratively co-registered to their mean, using a 6 degrees-of-freedom, followed by 9 degrees-of-freedom rigid body registration using SPM12. Next, image intensity histograms across each participant’s time series of images were normalized using an in-house developed differential bias correction algorithm,^28^ and the SyN diffeomorphic registration algorithm from ANTs software^24^ was used to compute deformations between each pair of images in each direction, producing the Jacobian determinant images and the ‘annualized’ log of the Jacobian determinant from the deformation in each direction. The voxel values of the Jacobian determinant image represent the expansion or contraction of each voxel over time, and those of the annualized log Jacobian determinant image can be thought of as analogous to an annualized per cent change at each voxel. The SyN deformations were applied in each direction, respectively, to the original bias corrected late and early images, to get the early image warped to the late, and the late image warped to the early image, and average them with their respective originals, resulting in a ‘synthetic late image’ and a ‘synthetic early image’. Then, we segmented the synthetic early and late images each using the same segmentation pipeline described previously.

To assess the cross-sectional and longitudinal atrophy at the voxel level, the baseline tissue-class segmented images and the annualized log of the Jacobian determinant for each voxel in each participant were smoothed with a 6 mm full-width at half maximum Gaussian smoothing kernel. Between-group voxel-based comparisons were computed using SPM12 and displayed after correcting for multiple comparisons with false discovery rate (FDR; *P* < 0.05). Our main analysis was voxel-based analysis to investigate group differences. However, because our secondary objective was to determine whether the rates of grey matter atrophy are associated with clinical disease progression in MCI-LB, we employed a secondary atlas-based ROI analysis. Data from the atlas-based ROI analysis was utilized for investigating the associations between atrophy rates and clinical disease progression.

### Pathologic examination

We used standardized methods for the neuropathologic assessment by expert neuropathologists (MEM, DWD and RRR) blinded to MRI results. Sampling was done according to the CERAD protocol^29^ and the fourth report of the DLB Consortium.^17^ Immunohistochemistry to detect Lewy-related pathology was performed using monoclonal antibody to alpha-synuclein (LB509; 1:200; Zymed, San Francisco, CA, USA) using a protocol (formic acid pre-treatment and DAKO DAB polymer signal detection) as previous described.^30^ The presence, density, semiquantitative scores and distribution of Lewy body-related pathology were based on recommendations of the fourth report of the DLB Consortium.^17^ Amyloid-beta plaques and neurofibrillary tangles were staged according to the National Institute on Aging-Alzheimer’s Association (NIA-AA) criteria.^31,32^

Cases with intermediate or high likelihood DLB (according to the fourth report of the DLB Consortium Criteria) who had low Alzheimer’s disease pathology (according to the NIA-AA criteria) were classified as Lewy body disease; cases with intermediate or high likelihood DLB and intermediate or high Alzheimer’s disease were classified as having mixed Lewy body disease–Alzheimer’s disease pathology and cases with intermediate or high Alzheimer’s disease and no Lewy body disease pathology or low likelihood DLB were classified as Alzheimer’s disease.

### Statistical analysis

Baseline demographic and clinical characteristics were summarized with means and standard deviations for continuous variables, and with counts and percentages for categorical variables. Baseline characteristics were compared between MCI-LB and controls using conditional logistic regression models to account for the matching. MMSE and CDR–SOB scores had fitting problems resulting in lack of convergence, and in those instances the comparisons came from an exact conditional logistic model. The differences in the characteristics of MCI-LB stables and MCI-LB progressors were analysed with *t*-tests for continuous variables and *χ*^2^ tests for categorical variables. Assessments of atlas-based ROI findings were corrected for multiple comparisons by requiring the corrected FDR to be *P* < 0.05. Findings from atlas-based ROI analysis were used to determine regions used for partial Pearson correlation analysis with CDR–SOB. These correlations were adjusted for age.

### Data availability

The Mayo Clinic Study of Aging and Alzheimer’s Disease Research Center make data available to qualified researchers upon reasonable request.

## Results

### Characteristics of the cohort

Baseline demographic and clinical characteristics of MCI-LB and CU participants and subgroups of MCI-LB stables and MCI-LB progressors are listed in Table 1. MCI-LB patients did not differ in age and sex from the CU controls, owing to matching. The median time interval between serial MRI sessions was 2.2 years for CU and 1.3 years for MCI-LB (1.4 years for MCI-LB stables and 1.3 years for MCI-LB progressors) due to the differences in follow-up time intervals among the MCSA and ADRC cohorts. To account for the longer MRI time intervals in CU compared with MCI-LB patients, all longitudinal measurements were annualized. Higher CDR–SOB (*P* < 0.001), UPDRS-III (*P* < 0.001), and lower MMSE scores (*P* < 0.001), and a higher frequency of *APOE* ε4 carriers (*P* = 0.029) were observed in the MCI-LB group compared with the CU at baseline.

**Table 1:** Characteristics of participants

	CU (*n* = 112)	MCI-LB (*n* = 56)	*P*-value*	MCI-LB stable (*n* = 28)	MCI-LB progressor (*n* = 28)	*P*-value**
Age, years	70.5 (7.1)	70.5 (7.1)	0.99	70.8 (7.8)	70.1 (6.4)	0.73
Males, no. (%)	106 (95%)	53 (95%)	1.00	26 (93%)	27 (96%)	0.55
APOE ε4, no. (%)	21 (19%)	19 (35%)	0.029*	7 (26%)	12 (43%)	0.19
Education, years	15.3 (2.7)	16.1 (2.6)	0.093	16.2 (2.4)	16.0 (2.8)	0.76
Scan interval, years	2.2 (0.8)	1.3 (0.9)	<0.001*	1.4 (1.1)	1.3 (0.5)	0.61
MMSE	28.4 (1.2)	26.6 (2.5)	<0.001*	27.5 (1.8)	25.7 (2.8)	0.005**
CDR–Sum of Boxes	0.0 (0.2)	1.7 (0.9)	<0.001†	1.3 (0.8)	2.1 (0.9)	<0.001**
UPDRS-III	0.7 (2.2)	6.2 (4.4)	<0.001*	4.7 (4.5)	7.5 (3.9)	0.016**
Visual hallucinations, no. (%)	—	13 (24%)		3 (11%)	10 (36%)	0.032**
Fluctuations, no. (%)	—	22 (40%)		6 (22%)	16 (57%)	0.008**
Parkinsonism, no. (%)	—	44 (80%)		18 (67%)	26 (93%)	0.015**
pRBD, no. (%)	—	49 (89%)		24 (89%)	25 (89%)	0.96
Cognitive impairment, years	—	5.3 (3.7)		5.4 (3.9)	5.2 (3.5)	0.84
Cholinesterase, no. (%)	—	46 (82%)		22 (79%)	24 (86%)	0.49
DLB features						
One, no. (%)	—	12 (21%)		9 (32%)	3 (11%)	0.002
Two, no. (%)	—	20 (36%)		14 (50%)	6 (21%)	
Three, no. (%)	—	18 (32%)		4 (14%)	14 (50%)	
Four, no. (%)	—	6 (11%)		1 (4%)	5 (18%)	

Mean (SD) listed for the continuous variables and count (%) for the categorical variables.

* *P*-values come from a conditional logistic model.

† The *P*-value is from an exact conditional logistic model.

** *P*-values come from a *t*-test for the continuous variables or a *χ*^2^ test for the categorical variables.

Characteristics of MCI-LB stables were similar to the MCI-LB progressors in age, sex, education and *APOE* ε4 status at baseline and scan time interval at follow-up. Compared with MCI-LB stables, MCI-LB progressors were clinically more impaired at baseline with lower MMSE scores (*P* =  0.005), higher CDR–SOB scores (*P* < 0.001), UPDRS-III scores (*P* =  0.016) and had a higher number of DLB core clinical features. The frequency of visual hallucinations, fluctuations and parkinsonism were higher in the MCI-LB progressors compared with the MCI-LB stables, while the frequency of pRBD was the same (89%) among the MCI-LB stables and MCI-LB progressors. Of the 56 MCI-LB participants, 46 (82%) were taking cholinesterase inhibitor treatments during follow-up. There were no differences in in anticholinesterase use among MCI-LB stables (79%) and MCI-LB progressors (86%; *P* = 0.49).

### Cortical atrophy at baseline

**Figure 1:**
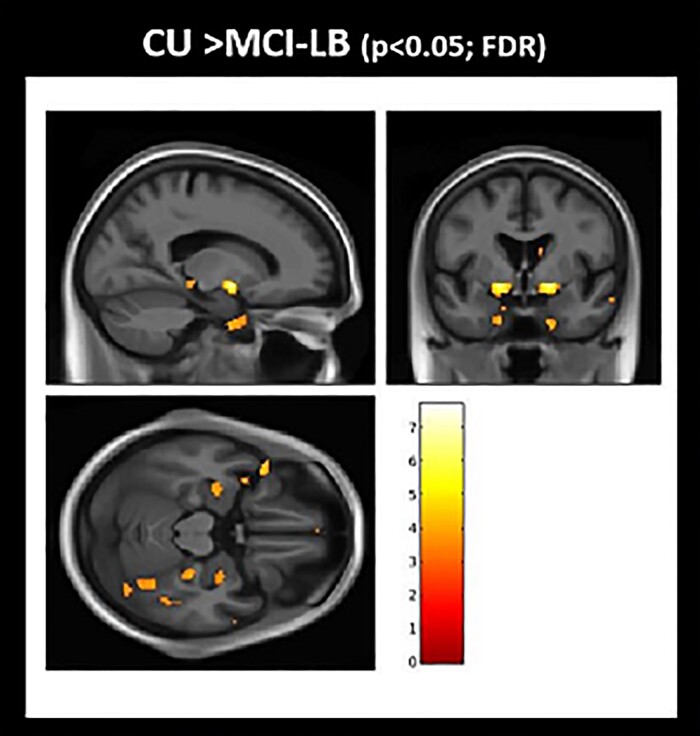
**Grey matter atrophy in MCI-LB at baseline: voxel-based analysis**. Voxel-based analysis show differences in grey matter atrophy in MCI-LB compared to CU projected to the 3D brain surface. Greater atrophy in the basal forebrain, inferior temporal and amygdala regions are seen in MCI-LB compared with the CU group (FDR; *P* < 0.05). *T*-score bar indicate magnitude of the differences.

Voxel-based analysis shows the pattern of grey matter atrophy at baseline comparing MCI-LB and CU (Fig. 1). The atrophy in MCI-LB was observed in the amygdala, inferior temporal lobe and basal forebrain region that localized to the nucleus basalis of Meynert. Notably, nucleus basalis of Meynert bilaterally was the most significant region of grey matter atrophy in MCI-LB compared with CU after correcting for multiple comparisons (FDR; *P* < 0.05). No differences in cortical thickness were found between MCI-LB stables and MCI-LB progressors at baseline. Neither did we find greater atrophy in CU compared with MCI-LB.

**Figure 2:**
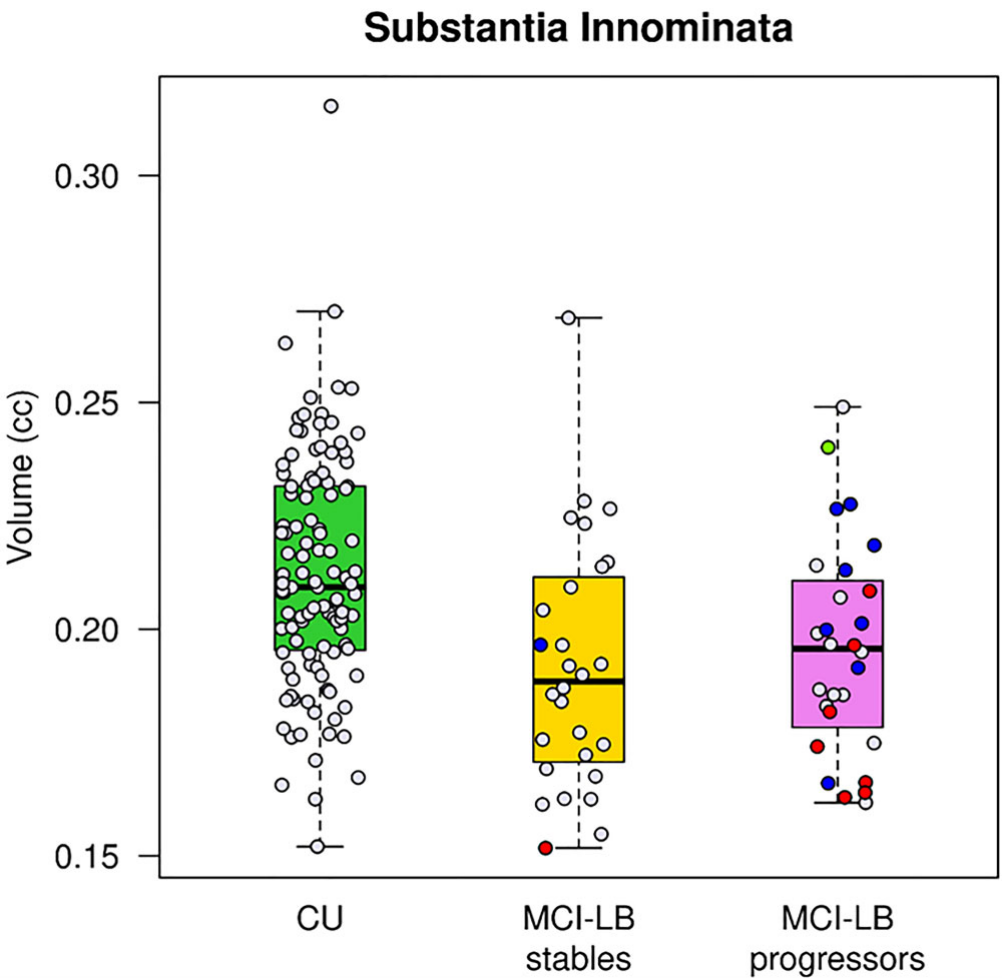
**Substantia innominata volumes**. Box plots show that MCI-LB stables (FDR corrected *P* =  0.007) and MCI-LB progressors (FDR corrected *P* =  0.028) had smaller substantia innominata volumes compared to CU. The pathologically confirmed cases were colour coded based on the pathologic diagnosis after a median (range) of 5.3 (2.4–10.6) years. Blue labels represent cases with intermediate or high likelihood DLB (according to the fourth report of the DLB Consortium Criteria) who had low Alzheimer’s disease pathology (according to the NIA-AA criteria) classified as Lewy body disease. Red labels represent cases with intermediate or high likelihood DLB and intermediate or high Alzheimer’s disease, classified as having mixed Lewy body disease–Alzheimer’s disease pathology. The single green label represents a case with high Alzheimer’s disease and no Lewy body disease pathology classified as Alzheimer’s disease.

Atlas-based ROI differences between MCI-LB and CU are shown in Supplementary Fig. 1. Consistent with the voxel-based analysis, the greatest grey matter atrophy in MCI-LB patients was observed in the substantia innominata ROI which includes the nucleus basalis of Meynert compared to CU (FDR corrected *P* =  0.001). In addition, grey matter atrophy included the amygdala, fusiform, lateral parietal precuneus, supramarginal angular, entorhinal and parahippocampal cortices in MCI-LB compared with CU (FDR corrected *P* < 0.05). Because several regions were involved, we grouped the differences based on their magnitude into the three levels thresholded as shown with a colour scale in Supplementary Fig. 1. No differences in cortical thickness were found between MCI-LB stables and MCI-LB progressors at baseline using atlas-based analysis (FDR corrected *P* > 0.05). The box plots in Fig. 2 displays the substantia innominata volumes in CU, MCI-LB stables and MCI-LB progressors. Both MCI-LB stables (FDR corrected *P* =  0.007) and MCI-LB progressors (FDR corrected *P* =  0.028) had smaller substantia innominata volumes compared with CU.

### Longitudinal rates of atrophy

**Figure 3:**
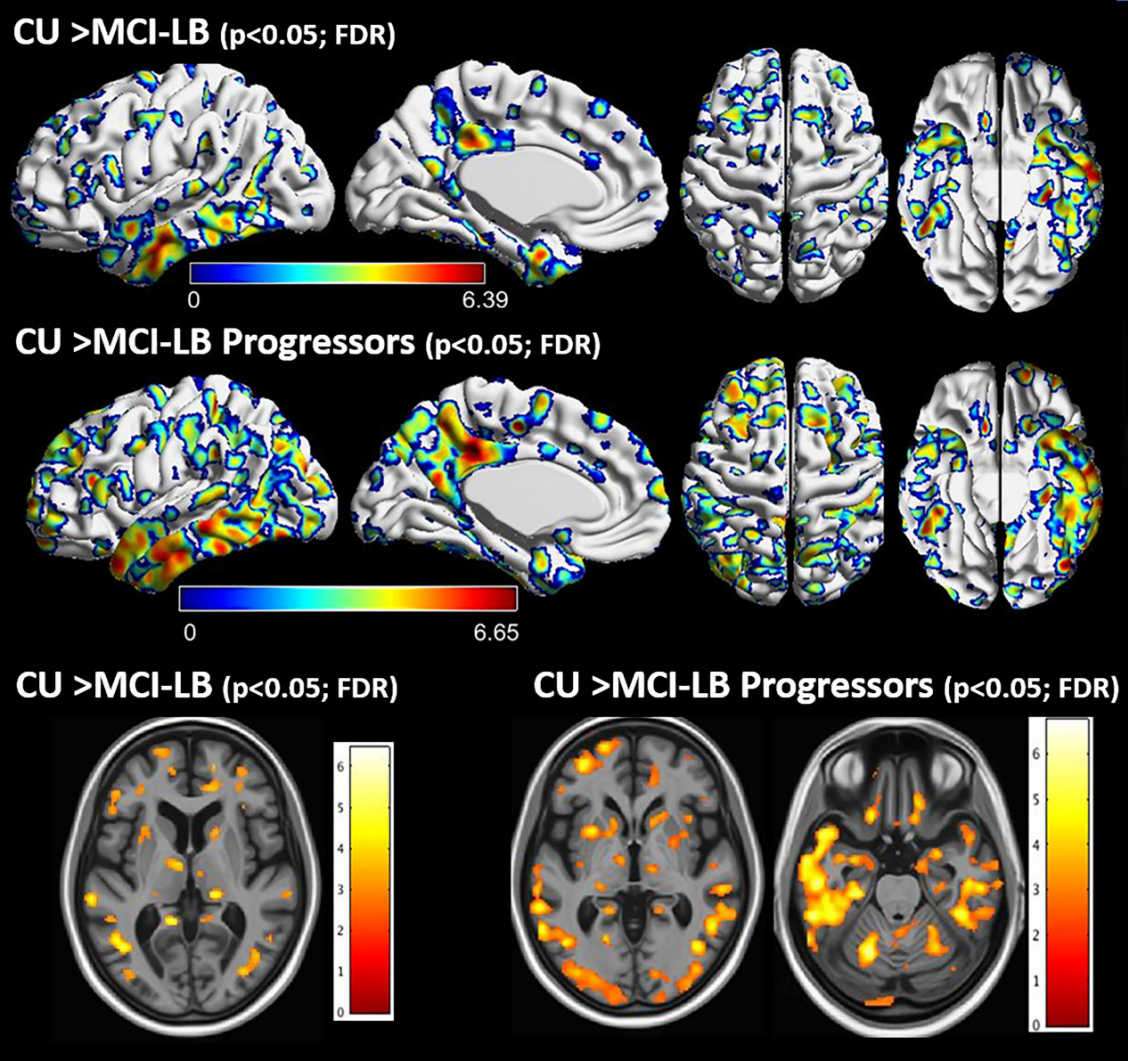
**Regional pattern of group differences in longitudinal rates of cortical atrophy: voxel-based analysis.** Voxel-based analyses show differences in the annualized rates of grey matter atrophy in MCI-LB compared to CU projected to the 3D brain surface. Greater rates of atrophy in the lateral and inferior temporal cortices and temporal pole, as well as medial and lateral parietal cortices is observed in MCI-LB compared with CU in the upper row, and in MCI-LB progressors compared with CU in the middle row (FDR; *P* < 0.05). Individual sections demonstrating differences in longitudinal rates of atrophy in the thalamus, amygdala and insula is shown in the bottom row. The blue colours around the FDR corrected *P* < 0.05 clusters are due to interpolation of edge voxels. *T*-score bars indicate magnitude of the differences.

Greater rates of longitudinal grey matter atrophy were observed in the lateral and inferior temporal cortices and temporal pole, as well as medial and lateral parietal cortices, thalamus and insula in both the MCI-LB and the DLB-progressor groups compared with CU on voxel-based analysis (Fig. 3). No differences in the annualized atrophy rates were observed between MCI-LB stables and CU or between MCI-LB stables and MCI-LB progressors on voxel-based analysis. Neither did we find greater atrophy in CU compared with MCI-LB.

Atlas-based ROI differences in rates of grey matter atrophy between MCI-LB patients and CU, and between MCI-LB progressors and CU, are shown in Supplementary Fig. 2. Consistent with voxel-based analysis, MCI-LB patients showed greater rates of grey matter atrophy in the temporoparietal cortices and these changes were more pronounced in those who progressed to DLB. Patients in the MCI-LB group showed the greatest rates of grey matter atrophy in the entorhinal and parahippocampal cortices, inferior and middle temporal cortices (FDR corrected *P* < 0.001) and also greater rates of grey matter atrophy in the posterior cingulate, precuneus, fusiform, supramarginal, angular, temporal pole, superior temporal, superior and middle frontal, lateral parietal and insular cortices, as well as thalamus, compared with CU (FDR corrected *P* < 0.05). Similarly, MCI-LB progressors showed greater rates of cortical atrophy in the inferior, middle and superior temporal, temporal pole, precuneus, supramarginal, angular, entorhinal and parahippocampal, lateral parietal, fusiform, and posterior cingulate cortex and thalamus (FDR corrected *P* < 0.001), as well as greater rates of atrophy in the lateral occipital, superior and middle frontal, insula, sensory motor, cuneus cortices, hippocampus, amygdala and caudate (FDR corrected *P* < 0.05), compared with CU. The only region that showed greater rates of atrophy in MCI-LB stables compared with CU was the entorhinal and parahippocampal cortices (FDR corrected *P* =  0.014). No difference in rates of atrophy was found between the MCI-LB stables and MCI-LB progressors after FDR correction for multiple comparisons (Supplementary Fig. 3).

### Correlation with disease progression

**Figure 4:**
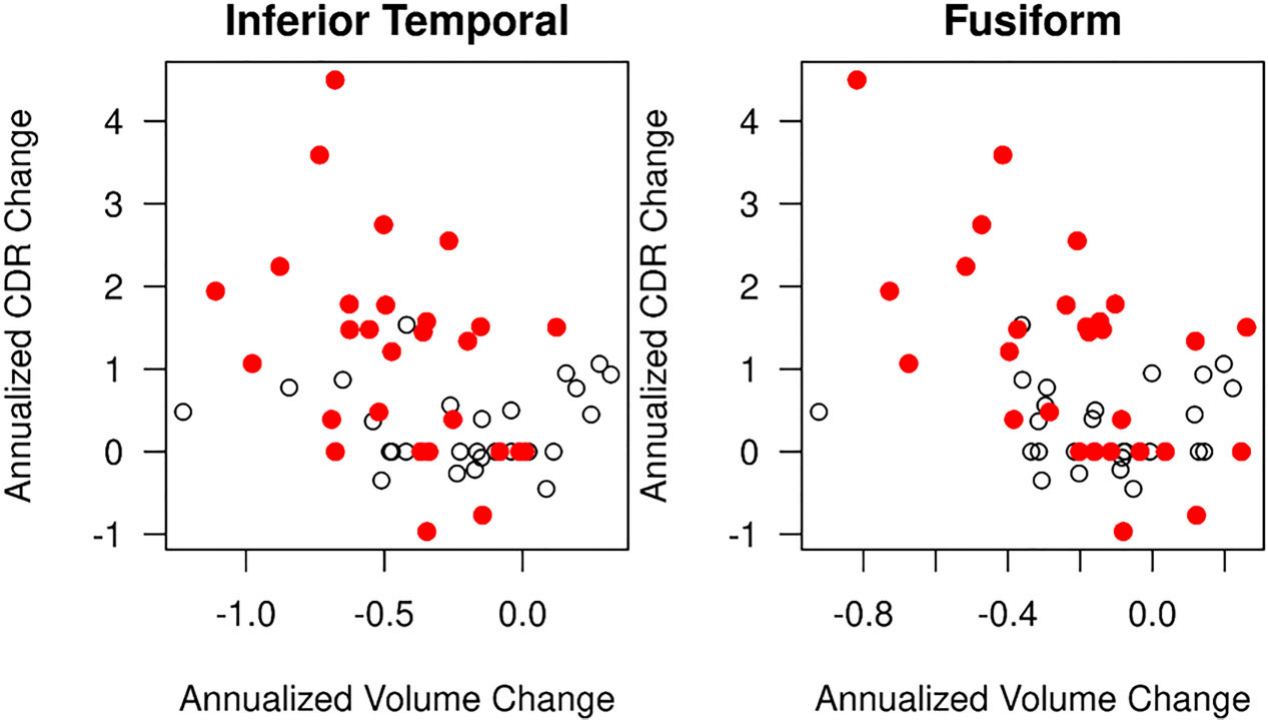
**Longitudinal decline in inferior temporal and fusiform gyrus grey matter correlates with clinical disease progression in MCI-LB**. In MCI-LB, longitudinal increase in CDR–SOB correlated with longitudinal rates of volume loss in the inferior temporal (*r* = −0.35; *P* =  0.011) and fusiform (*r* = −0.44; *P* < 0.001) cortices after adjusting for age at baseline. The open circles represent MCI-LB stables and the red circles represent MCI-LB progressors.

Among the ROIs with greater atrophy rates in MCI-LB compared with CU, decline in cortical volumes correlated with disease progression in only two of the ROIs (inferior temporal and fusiform). Correlations of longitudinal change in CDR–SOB and longitudinal change in cortical volumes are listed in Supplementary Table 1. In MCI-LB, longitudinal increase in CDR–SOB correlated with longitudinal rates of volume loss in the inferior temporal (*r* = −0.35; *P* =  0.011) and fusiform (*r* = −0.44; *P* < 0.001) cortices after adjusting for age at baseline (Fig. 4). Number of core clinical features at baseline was not associated with the rates of atrophy in any of the ROIs in MCI-LB (*P* > 0.05).

### Pathologic confirmation

Pathologic diagnosis was available in the autopsied MCI-LB cases (*n* = 18). Cases were classified either as Lewy body disease (*n* = 8; MCI-LB stable *n* = 1; MCI-LB progressor *n* = 7) or Lewy body disease–Alzheimer’s disease (*n* = 9; MCI-LB stable *n* = 1; MCI-LB progressor *n* = 8). One of the MCI-LB patients with parkinsonism and RBD at the time of baseline MRI progressed to probable DLB during follow-up and was found to have high likelihood Alzheimer’s disease and no Lewy body disease at autopsy. Thus, 17 of the 18 (94%) pathologically confirmed cases had Lewy body disease. The median (range) time from baseline MRI to death was 5.3 (2.4–10.6) years. Because of the wide range and variability in time from baseline MRI to death, pathologic diagnosis may not fully represent the extent of pathology at the time of baseline MRI. Therefore, we did not investigate the MRI findings in association with pathologic diagnosis. However, we present the substantia innominata volumes of the pathologically confirmed cases in Fig. 2 using diagnosis-based colour codes. Interestingly, the MCI-LB patient who was pathologically classified as Alzheimer’s disease had one of the largest substantia innominata volumes among the MCI-LB patients.

## Discussion

In this longitudinal structural MRI study, we demonstrated a baseline and longitudinal pattern of atrophy in MCI patients with one or more core DLB features (i.e. MCI-LB) and in those who progressed to probable DLB (i.e. MCI-LB progressors) at follow-up. At baseline, we found grey matter atrophy in the nucleus basalis of Meynert in MCI-LB compared with CU. Longitudinally, increased rates of grey matter atrophy were widespread in both cortical and subcortical grey matter with the greatest rates of atrophy in the entorhinal and parahippocampal gyri, temporoparietal association cortices, thalamus and the basal ganglia compared with CU. This pattern was most pronounced for the MCI-LB progressors, and greater rates of atrophy were associated with greater rates of clinical progression.

At baseline, MCI-LB patients had atrophy in the basal forebrain and specifically in the cholinergic nucleus basalis of Meynert. Inferior and medial temporal and parietal cortices were involved to a lesser extent but did not statistically differ from CU on voxel-based analysis after correction for multiple comparisons. Nucleus basalis of Meynert consists primarily of large acetylcholinesterase-positive neurons that innervate the cerebral cortex. Early LB-related pathology involves the basal forebrain cholinergic nuclei^33^ with significant loss of cholinergic neurons particularly in the nucleus basalis of Meynert.^33,34^ Atrophy in the substantia innominata region that includes the nucleus basalis of Meynert has been demonstrated in DLB and Alzheimer’s disease patients, but this was more prominent in patients with DLB.^11,35,36^ Substantia innominata atrophy was also reported in patients with mild probable DLB who were likely at the early stages of DLB and in MCI-LB.^8,37^Moreover, substantia innominata atrophy on MRI was associated with a better response to cholinesterase inhibitors in both patients with DLB and Alzheimer’s disease.^38^ Our findings provide evidence of neurodegeneration in the nucleus basalis of Meynert in prodromal DLB. We observed smaller volumes in the substantia innominata region both in MCI-LB stables and MCI-LB progressors and did not observe differences in grey matter atrophy at baseline between these two groups. Furthermore, seventeen of the eighteen (94%) MCI-LB patients had pathologically confirmed Lewy body disease at autopsy. Therefore, atrophy of the nucleus basalis of Meynert appears to be a feature of MCI-LB regardless of proximity to progression to probable DLB. These findings support the concept that cholinergic deficit plays an important role in prodromal DLB.

Although atrophy in the temporal and parietal cortical regions in MCI-LB were not statistically significant after correction for multiple comparisons on voxel-based analysis, there was evidence of smaller amygdala, inferior temporal and parietal cortex volumes in MCI-LB compared to CU using atlas-based ROI analysis evaluating 28 cortical and subcortical regions, even after correcting for multiple comparisons. This difference in findings using different analytic approaches may be due to the relatively small sample size and diminished power to detect smaller differences using voxel-based analysis versus atlas-based ROI analysis.

When investigating atrophy in patients with probable DLB, it is important to consider that many patients with Lewy body disease or probable DLB have additional Alzheimer’s disease pathology at autopsy.^39–41^ Amyloid-β and/or tau biomarker positivity was demonstrated in 61% of probable DLB patients from a large multinational cohort (*n* = 417).^42^ On the other hand, only 35% patients with MCI-LB showed amyloid-β positivity on PET,^43^ suggesting that amyloid-β pathology accumulates as MCI-LB patients progress to DLB and continues to increase thereafter.^44^ Amyloid-β deposition is associated with increased rates of atrophy,^13^ and tau pathology influences cortical volumes in probable DLB.^45^ Similarly, in MCI-LB, Alzheimer’s disease pathology may influence neurodegeneration in regions that are affected during the evolution of Alzheimer’s disease such as the nucleus basalis of Meynert and the medial temporal lobe, but the nucleus basalis of Meynert in Alzheimer’s disease is not affected until later in Alzheimer’s disease compared to DLB,^37^ and a more prominent atrophy in the nucleus basalis of Meynert region was observed in MCI-LB compared to MCI due to Alzheimer’s disease.^8^ We also note that the MCI-LB patient with pure Alzheimer’s disease pathology at autopsy had one of the most preserved substantia innominata volumes among MCI-LB. In addition, among the medial temporal lobe regions, we did not identify hippocampal atrophy in MCI-LB compared with CU. Hippocampal atrophy is a feature of additional co-occurring Alzheimer’s disease pathology in DLB,^19^ and hippocampal preservation in MCI predicts future progression to probable DLB versus Alzheimer’s disease dementia.^3,46^ Thus, preservation of hippocampal volumes in the MCI-LB patients we studied suggest that contribution of Alzheimer’s disease to neurodegeneration in MCI-LB was not substantial.

**Figure 5:**
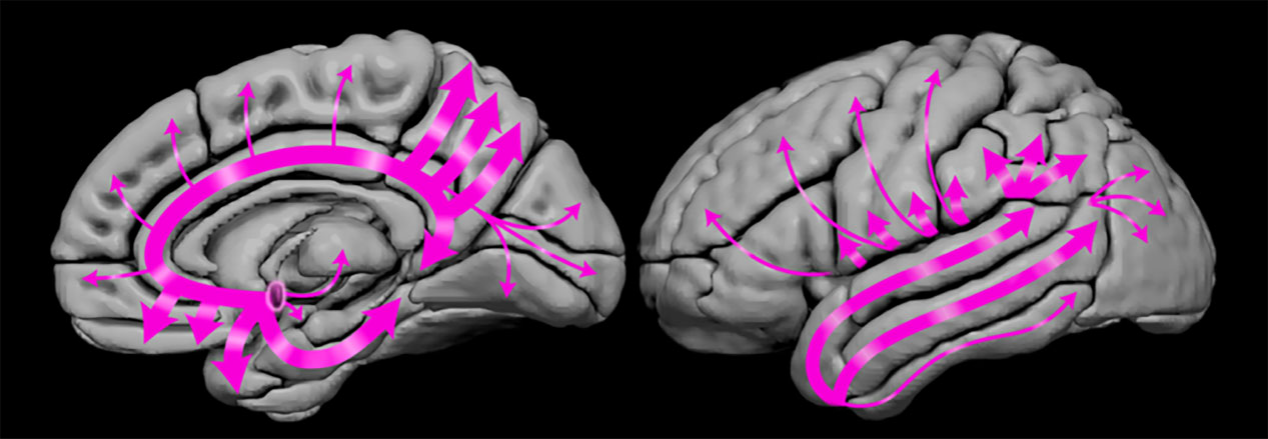
**Cholinergic projections from the nucleus basalis of Meynert.** Schematic showing the projections from nucleus basalis of Meynert to the cortex, amygdala, thalamus and caudate. Thicker arrows suggest more dense projections from this cholinergic nucleus to limbic and temporoparietal association cortices and the amygdala where the greatest rates of atrophy were observed in MCI-LB compared with CU.

Longitudinal analysis of serial MRIs demonstrated increased rates of atrophy in MCI-LB throughout the limbic and the neocortical regions, particularly in the temporoparietal cortices and the insula. Atrophy in the insula has been reported in MCI-LB in cross-sectional studies, which likely included a subset of patients who later progressed to probable DLB.^5,6^ Increased rates of atrophy were pronounced in MCI-LB progressors, but they were only observed in the entorhinal and parahippocampal cortices in MCI-LB stables compared with CU, suggesting that atrophy rates in MCI-LB could be non-linear and accelerate as MCI-LB patients progress to probable DLB. Most cortical and subcortical regions with greater rates of atrophy were the brain regions that receive dense cholinergic inputs from the nucleus basalis of Meynert^47^ such as the amygdala and the temporal and parietal association cortex as demonstrated in Fig. 5. Hence, degeneration of cholinergic neurons of the nucleus basalis of Meynert at baseline and associated cholinergic denervation may have contributed to increased atrophy rates in the limbic and neocortical regions in MCI-LB. We did not observe increased rates of atrophy in the substantia innominata region. It is possible that atrophy of nucleus basalis of Meynert occurs very early during the evolution of Lewy body disease and reaches a plateau at the symptomatic stages such as in MCI-LB.

Consistent with the widespread increased rates of atrophy in MCI-LB progressors but not in MCI-LB stables, higher rates of atrophy in the inferior temporal and fusiform gyri were associated with greater increase (worsening) in CDR–SOB scores. Cortical atrophy has been associated with clinical disease progression in probable DLB,^9^ and here we demonstrate a similar finding in MCI-LB localized to the inferior temporal lobe. Inferior temporal lobe is impacted by both α-synuclein and neurofibrillary tangle tau pathologies in transitional and diffuse Lewy body disease.^48^ Increased tau deposition in the inferior temporal lobes at much higher levels than the medial temporal lobe regions has been demonstrated on PET in probable DLB.^49,50^ Altogether, these findings suggest that the inferior temporal lobe may be particularly vulnerable to neurodegeneration either due to α-synuclein or neurofibrillary tangle tau pathologies, or both early in the course of DLB with accelerated atrophy rates and associated clinical progression in MCI-LB.

A strength of this study is that we measured the baseline cross-sectional and longitudinal rates of atrophy in MCI-LB who either progressed to probable DLB or remained as MCI-LB during a similar length of follow-up. Findings in the MCI-LB stable and progressor groups reveal the time course of neurodegeneration in prodromal DLB demonstrating that neurodegeneration is associated with clinical progression with slower rates of atrophy during a stable clinical course and accelerated rates of atrophy as MCI-LB patients progress to dementia. This study also has several limitations. First, the sample sizes were modest, which may be one of the reasons for relatively weaker findings using voxel-based analysis compared to atlas-based ROI analysis. Second, to keep the follow-up interval consistent between the MCI-DLB patients who remained stable and progressed to DLB, the last two evaluations prior to and at the time of progression to DLB were assessed for MCI-LB progressors, while the last two evaluations were considered for MCI-LB stables. As reported in Table 1, baseline clinical characteristics of MCI-LB stable and MCI-LB progressor groups were different with greater disease severity observed in MCI-LB progressors. Although we did not find statistically significant differences in the rates of atrophy between MCI-LB stables and MCI-LB progressors, disease severity could have potentially influenced the higher number of regions impacted in MCI-progressors than MCI-LB stables. Third, we assumed linearity in the rates of change when annualizing the longitudinal measurements that occurred within 1–2 years, which may have introduced error when comparing groups with different follow-up intervals. Finally, we did not have autopsy confirmation of Lewy body disease and presence of additional Alzheimer’s disease pathology in the entire cohort, and the pathologic examination occurred many years after the baseline MRI making it difficult to associate MRI findings with pathologic diagnosis. Therefore, it is not possible to attribute findings in MCI-LB to Lewy body disease and/or Alzheimer’s disease pathology. Investigations into the prevalence of and Alzheimer’s disease biomarkers and their contribution to neurodegeneration in prodromal DLB is warranted.

MCI-LB is characterized by atrophy in the nucleus basalis of Meynert, making neurodegeneration in the cholinergic system a prominent feature of MCI-LB. Grey matter atrophy progresses in alignment with clinical disease progression in MCI-LB with accelerated rates of atrophy observed in MCI-LB patients who progress to probable DLB within approximately a year. Although additional Alzheimer’s disease pathology may be responsible for accelerated neurodegeneration during the progression of MCI-LB patients to dementia, the cholinergic deficits at baseline may also be contributing to the widespread accelerated atrophy rates in MCI-LB. Patients with probable DLB, particularly those with negative Alzheimer’s disease biomarkers show the best response to cholinesterase inhibitors.^51^ Because neurodegeneration in the nucleus basalis of Meynert is a characteristic of MCI-LB, patients with MCI-LB should be considered as candidates for cholinesterase inhibitor treatment. An intriguing possibility to examine is whether such treatment given during the prodromal stage of DLB serves to slow the neurodegeneration that is due to cholinergic denervation.

## Supplementary Material

fcac013_Supplementary_Data

## Abbreviations

ADRC =: Alzheimer’s Disease Research Center
CDR =: Clinical Dementia Rating Scale
CU =: cognitively unimpaired
DLB =: dementia with Lewy bodies
MCALT =: Mayo Clinic Adult Lifespan Template
MCI-LB =: mild cognitive impairment with core clinical features of DLB
MCSA =: Mayo Clinic Study of Aging
pRBD =: probable rapid eye movement sleep behaviour disorder
SPM =: statistical parametric mapping
TBM-Syn =: tensor-based morphometry with symmetric normalization
UPDRS-III =: Unified Parkinson’s Disease Rating Scale part III
