# Supplementary material for: Longitudinal atrophy in prodromal dementia with Lewy bodies points to cholinergic degeneration
Source: Brain Commun. 2022 Feb 7;4(2):fcac013. doi: 10.1093/braincomms/fcac013 (PMC8994111; doi:10.1093/braincomms/fcac013)
Supplement: fcac013_Supplementary_Data [file fcac013_supplementary_data.pdf]

SUPPLEMENTAL MATERIAL

Supplemental Figure 1: Gray matter atrophy in MCI-LB at baseline: atlas-based region of interest analysis: Differences in atrophy across 28 cortical and subcortical gray matter regions are corrected for multiple comparisons using FDR on the regional level. Individual p-values are thresholded into 3 levels represented by the following color scale: grey for non-significant; light purple for p value between 0.01 to 0.05 with FDR correction; dark purple for p value less than 0.01 with FDR correction. The greatest differences in atrophy was observed in the substantia innominata, temporal and parietal association cortices. The bar graph demonstrates reduced substantia innominata volumes in the MCI-LB stables and MCI-LB progressors compared to CU.

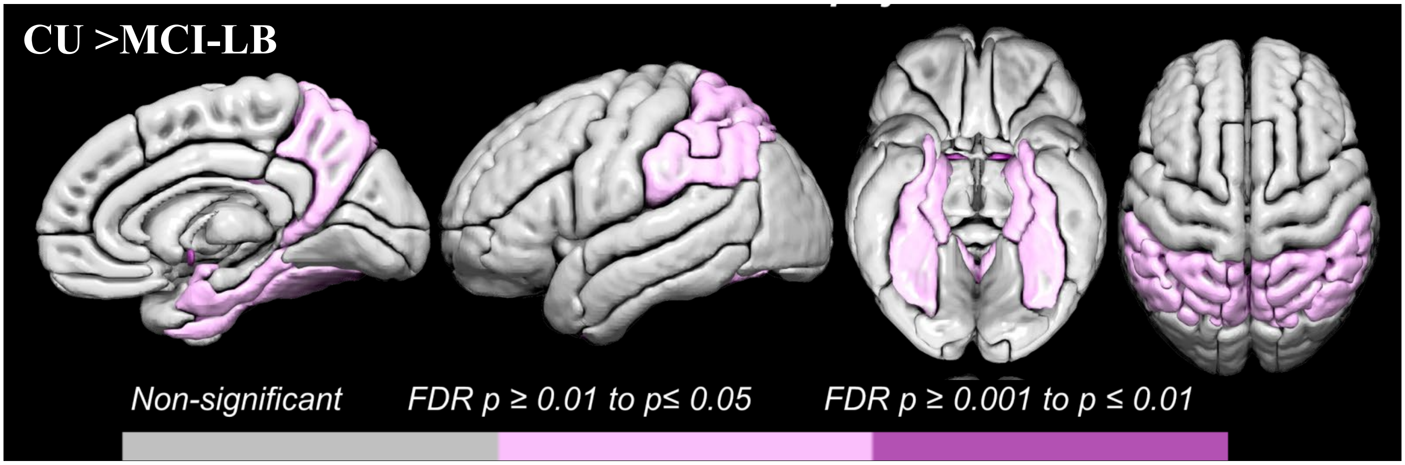

Supplemental Figure 2: Regional pattern of group differences in longitudinal rates of gray matter atrophy on atlas-based region of interest analysis: Differences in atrophy rates across 28 cortical and subcortical gray matter regions are corrected for multiple comparisons using FDR on the regional level. Individual p-values were thresholded into 3 levels represented by color scale: gray for non-significant; light purple for p value between 0.01 to 0.05 with FDR correction; darker purple for p value less than 0.01 with FDR correction, and darkest purple for p value less than 0.001 with FDR correction. Although atrophy rates greater than CU were widespread in both the MCI-LB and MCI-LB progressor groups, the greatest atrophy rates were observed in the entorhinal and parahippocampal gyri, as well as the temporal and parietal association cortices.

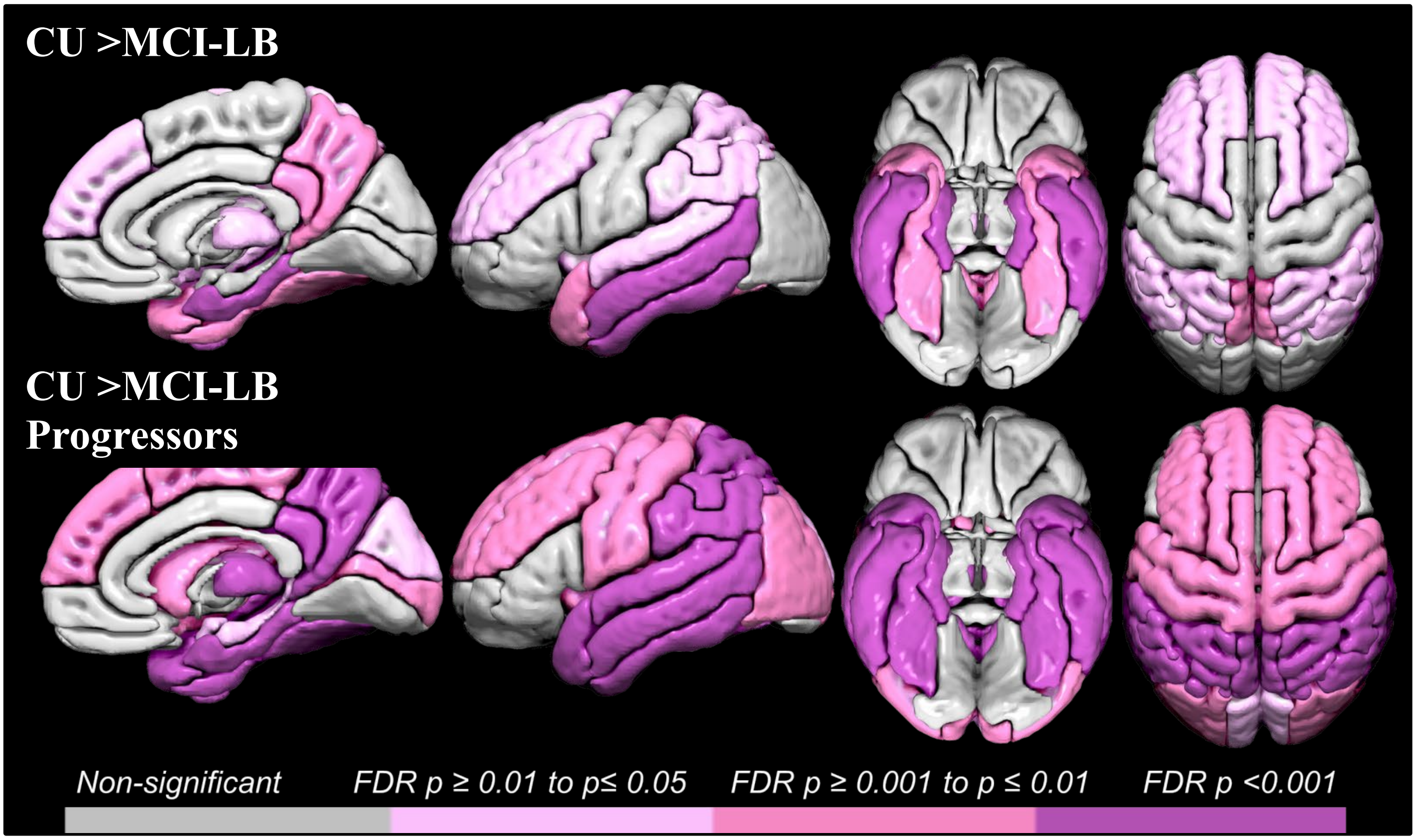

Supplemental Figure 3: Between group comparison of the rates of atrophy among in MCI-LB stables and MCI-LB progressors and cognitively unimpaired (CU) controls with and without FDR correction for multiple comparisons. The ranking from top to bottom is based on the statistical significance of the difference.

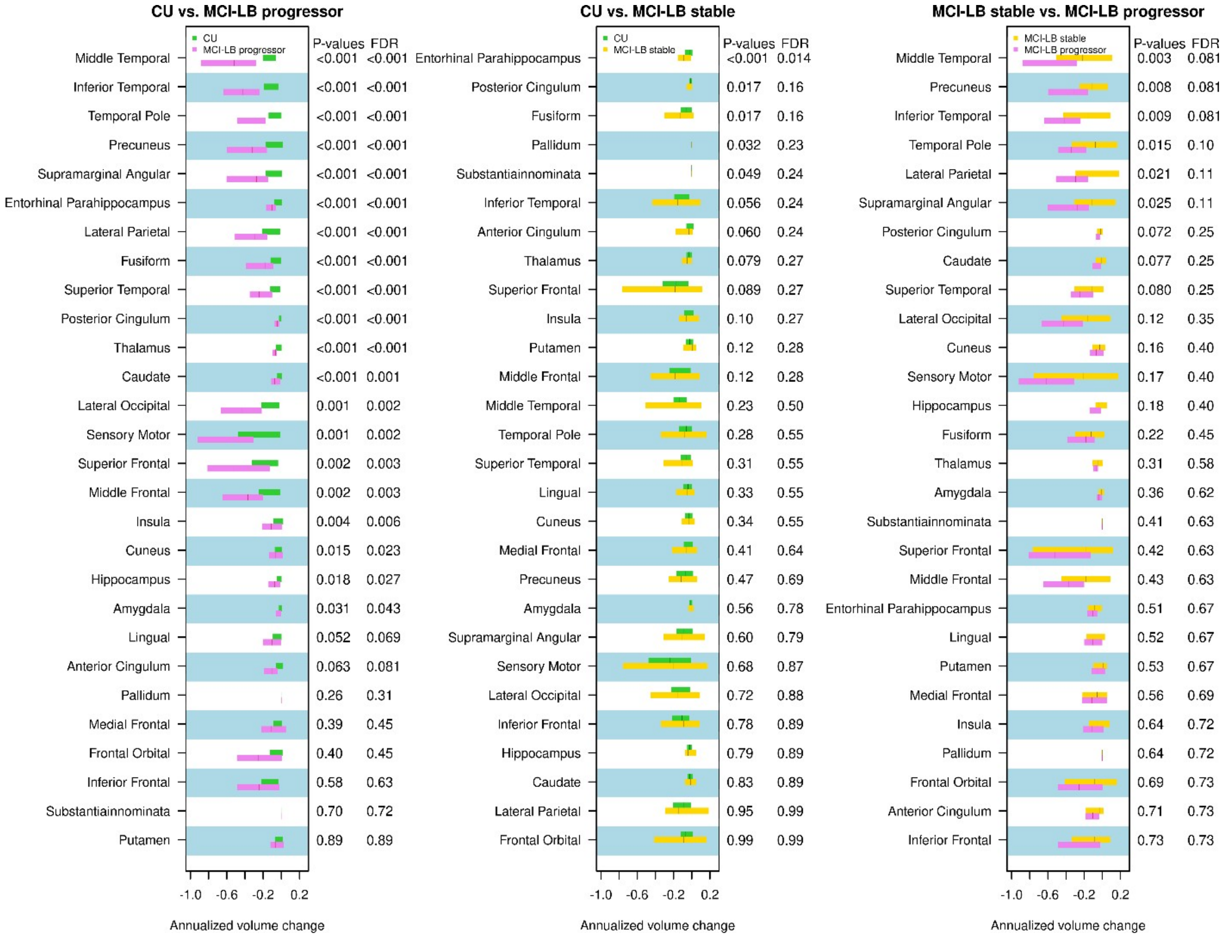

Supplemental Table 1: Correlations of annualized volumetric changes on atlas-based region of interest analysis with annualized changes in CDR sum of boxes. Partial Pearson correlations (95% confidence interval) adjusted for baseline age with an FDR correction for multiple comparisons.

	Correlation (95% CI)	P-value	FDR
Amygdala	0.182 (-0.087, 0.427)	0.19	0.61
Putamen	0.148 (-0.122, 0.398)	0.28	0.61
Cuneus	0.099 (-0.171, 0.355)	0.48	0.72
Thalamus	0.082 (-0.187, 0.34)	0.56	0.77
Lateral Occipital	0.07 (-0.199, 0.329)	0.62	0.77
Posterior Cingulum	0.033 (-0.235, 0.295)	0.82	0.88
Pallidum	0.009 (-0.257, 0.274)	0.95	0.95
Lateral Parietal	-0.023 (-0.286, 0.244)	0.87	0.90
Lingual	-0.039 (-0.301, 0.228)	0.78	0.87
Substantia Innominata	-0.04 (-0.302, 0.228)	0.78	0.87
Supramarginal Angular	-0.066 (-0.326, 0.203)	0.63	0.77
Middle Frontal	-0.077 (-0.335, 0.192)	0.58	0.77
Superior Frontal	-0.096 (-0.353, 0.173)	0.49	0.72
Insula	-0.103 (-0.358, 0.167)	0.46	0.72
Medial Frontal	-0.111 (-0.365, 0.159)	0.42	0.72
Superior Temporal	-0.136 (-0.387, 0.134)	0.33	0.61
Precuneus	-0.142 (-0.393, 0.128)	0.31	0.61
Sensory Motor	-0.146 (-0.396, 0.124)	0.29	0.61
Caudate	-0.149 (-0.398, 0.121)	0.28	0.61
Inferior Frontal	-0.156 (-0.405, 0.114)	0.26	0.61
Temporal Pole	-0.157 (-0.406, 0.113)	0.26	0.61
Entorhinal Parahippocampus	-0.169 (-0.416, 0.101)	0.22	0.61
Frontal Orbital	-0.184 (-0.428, 0.085)	0.18	0.61
Anterior Cingulum	-0.237 (-0.473, 0.03)	0.084	0.47
Middle Temporal	-0.256 (-0.488, 0.01)	0.062	0.43
Hippocampus	-0.261 (-0.492, 0.005)	0.057	0.43
Inferior Temporal	-0.345 (-0.559, -0.087)	**0.011**	0.15
Fusiform	-0.437 (-0.63, -0.195)	**<0.001**	**0.026**
